# Personal Protective Equipment Alters Leg Muscle Fatigability Independent of Transcranial Direct Current Stimulation: A Comparison with Pre-COVID-19 Pandemic Results

**DOI:** 10.3390/brainsci11080962

**Published:** 2021-07-22

**Authors:** Alexandra C. Fietsam, Justin R. Deters, Craig D. Workman, Thorsten Rudroff

**Affiliations:** 1 Department of Health and Human Physiology, University of Iowa, Iowa City, IA 52242, USA; alexandra-fietsam@uiowa.edu (A.C.F.); justin-deters@uiowa.edu (J.R.D.); craig-workman@uiowa.edu (C.D.W.); 2 Department of Neurology, University of Iowa Health Clinics, Iowa City, IA 52242, USA

**Keywords:** tDCS, fatigue, leg muscles, brain, PPE, face mask

## Abstract

In response to the COVID-19 pandemic, the use of personal protective equipment (PPE; e.g., face mask) has increased. Mandating subjects to wear PPE during vigorous exercise might affect the fatigue outcomes of transcranial direct current stimulation (tDCS) studies. The purpose of this study was to investigate whether the use of PPE affected the performance of a tDCS-influenced fatigue task in healthy adults. A total of 16 young and healthy subjects were recruited and wore PPE during an isokinetic fatigue task in conjunction with sham, 2 mA, and 4 mA tDCS conditions. Subjects were matched to subjects who did not wear PPE during our previous pre-pandemic study in which right knee extensor fatigability increased under these same conditions. The results show that right knee extensor fatigability, derived from torque and work (FI-T and FI-W, respectively), was higher in the PPE study compared to the No PPE study in the sham condition. Additionally, there were no differences in knee extensor fatigability or muscle activity between sham, 2 mA, and 4 mA tDCS in the present study, which contrasts with our previous results. Thus, PPE worn by subjects and researchers might have a detrimental effect on fatigue outcomes in tDCS studies irrespective of the stimulation intervention.

## 1. Introduction

In response to the COVID-19 pandemic, the use of personal protective equipment (PPE; e.g., face mask, face shield) has risen to reduce the spread of the virus. In human subject research, wearing PPE during vigorous motor function tests is important to prevent the spread of infectious respiratory droplets [1]. However, the ability to perform motor function tests, such as fatiguing exercises, while wearing PPE is potentially problematic because it may alter the study outcomes by decreasing the amount of oxygen available and/or increasing air trapping, which may reduce carbon dioxide exchange [2]. The resulting hypercapnic hypoxia postulated by Chandrasekaran and Fernandes (2020) may induce an acidic environment in the alveoli or blood and increase cardiac overload, anaerobic metabolism, and renal overload [2], all of which might worsen the underlying pathologies of frequently studied chronic diseases.

Fatigue is a common study outcome and has been defined as “the decrease in physical and/or mental performance that results from changes in central, psychological, and/or peripheral factors” [3]. Furthermore, performance fatigability is the magnitude or rate of change in a performance criterion relative to a reference value over a given time of task performance, and perceptions of fatigue are the subjective sensations of weariness, increasing sense of effort, mismatch between effort expended and actual performance, or exhaustion [4]. Several researchers, including our laboratory, have investigated the effects of transcranial direct current stimulation (tDCS) on performance fatigability and the perception of fatigue in healthy subjects [5,6,7,8,9] and in people with neurological disorders [10,11,12,13,14,15,16,17]. Some of these studies reported improved performance fatigability after unilateral or bilateral tDCS [18,19,20], which may be a result of increased corticospinal excitability coupled with altered motor unit recruitment from stimulation [21]. However, some of these studies found no effect on fatigue [22,23], or even increased fatigue after tDCS [5,6,8]. Thus, tDCS fatigue outcomes are inconsistent, and the addition of PPE might introduce another source of variability and increase outcome ambiguity.

Muscle metabolism highly depends on the efficient exchange of O_2_ and CO_2_, which may be interrupted by wearing a face mask [24,25,26]. Breathing hypoxic air significantly increases muscle fatigue [27,28], particularly during strenuous exercise when the energy demand of working muscles is often not met by increased oxygen delivery, even without a mask. Additionally, the hypercapnic state potentially induced by mask wearing may increase feelings of claustrophobia and anxiety [29,30,31] and further exacerbate fatigue. However, it is unclear if wearing a mask can influence performance outcomes. For example, it has been previously shown that wearing PPE during exercise resulted in a reduction in maximal power [32], while others showed no effect of PPE on time to exhaustion during a cycle ergometry test [33], or distance walked during a 6 min walk test (6MWT) [26].

Our previous pre-pandemic studies [5,6] found that both 2 mA and 4 mA transcranial direct current stimulation over the left motor cortex (M1) increased fatigability of the right knee extensors in young, healthy adults. However, conducting research studies in which subjects are required to wear PPE during vigorous exercise might independently alter the fatigue outcomes of tDCS studies, making reproducibility and comparison with non-pandemic era studies problematic. Consistent with our previous protocols, the current study evaluated the effects of 2 mA and 4 mA M1 tDCS on the performance of an isokinetic fatigue test of the knee extensors in a sample of young, healthy subjects while the subjects wore PPE (i.e., face masks and face shields). We hypothesized that wearing PPE would significantly increase the subjects’ fatigue profile (i.e., greater decrease in torque during the fatigue task) compared to the data collected on subjects without PPE from our previous studies [5,6] independent of the tDCS intensity.

## 2. Materials and Methods

### 2.1. Subjects

A total of 16 young, healthy subjects were recruited for the current study. Subjects were matched and compared to the 16 young, healthy subjects from our previous study ([6]; see Table 1 for subject characteristics). The inclusion criteria for this study were: (1) between the age of 18 and 30 years old, (2) right side-dominant, (3) able to walk independently for 6 consecutive min, (4) participate in at least 30 min of moderate-intensity physical activity on at least 3 days of the week for at least the last 3 months, (5) currently not taking any psychoactive medications, and (6) without chronic neurological, psychiatric, or medical conditions. The exclusion criteria were: (1) previously testing positive for COVID-19, (2) pregnancy, (3) known holes or fissures in the skull, (4) the presence of metallic objects or implanted devices in the skull (e.g., metal plate), or (5) are a current student or under the direct supervision of the study personnel. The study was performed in accordance with the Declaration of Helsinki and was approved by the Institutional Review Board of The University of Iowa. All subjects provided written informed consent before participating in the study.

### 2.2. Study Design

Recruitment and experimental sessions were conducted from August 2020 to February 2021. During this time, new COVID-19 infections were at pandemic highs in the US, especially during the winter months, with a 7-day average of 220,000 new cases [34]. Many research labs in the US were either not allowed to conduct human subject studies or had to follow strict protective guidelines, such as wearing PPE by investigators and research participants. Thus, the timing and relevancy of this investigation were such that performing human subject studies in this pandemic environment included university-mandated PPE requirements. Therefore, a double-blind, randomized, sham-controlled crossover study design was applied for the present study and compared via a between-subject analysis of a similarly designed study [6]. Each subject completed a total of 4 visits to the laboratory, with each visit spaced ~1 week apart. The methods used in this study were identical to our previous study [6], except in the current study, the subjects wore a disposable 17.5 × 9.5 cm 3-ply face mask (the standard mask at our institution; Zhejiang Bada Sport Inc., Jinhua City, Zhejiang, China) and a plastic face shield throughout each visit, and SpO2 was measured during fatigue testing. During the initial visit, isokinetic and isometric strength testing was performed to establish leg dominance and to provide normalization data for EMG analysis. Only right side-dominant subjects participated in this study to avoid potential brain morphology differences between right and left side-dominant people [35]. To familiarize the subjects with the fatigue protocol employed in the subsequent sessions, the subjects also performed the isokinetic fatigue task (FT) in the first session. During visits 2–4, the FT was repeated in conjunction with 20 min of tDCS at one of three randomly assigned intensities (2 mA, 4 mA, or sham).

### 2.3. Isokinetic/Isometric Strength Testing

Strength testing and the FT were performed on a HUMAC NORM isokinetic dynamometer (CSMi, Stoughton, MA, USA). Strength testing began with a 15-repetition warm-up of the knee extensors and flexors at 60°/s (concentric/concentric (muscle shortening contraction)). After a 30 s rest, 3 sets of 1 maximal effort isometric (fixed position) contraction of the knee extensors and knee flexors were performed at 65° and 30°, respectively, with ≥30 s of rest between each set. Next, isokinetic (fixed speed) strength testing consisted of 5 sets of 1 maximal effort knee extension and flexion (60°/s, concentric/concentric). Again, at least 30 s of rest was provided between each set. All tests were performed on the right leg first and the left leg second. The largest torque obtained from a given muscle group in any of the strength testing (isometric or isokinetic) was used to verify leg dominance. Strong verbal encouragement was provided to help ensure maximal effort on each repetition.

### 2.4. Isokinetic Fatigue Task (FT)

The FT consisted of 40 consecutive maximal contractions of the knee extensors and flexors (120°/s, concentric/concentric). Sessions 2–4 began with the same 15-repetition isokinetic warm-up, as previously described. The right leg FT was always performed first, followed by the left leg; the FTs of both legs were completed in ≤5 min. Strong verbal encouragement and visual feedback (i.e., per rep work bars) were provided to encourage maximum effort throughout the FT. Peak torque and total work for each rep were retained for analysis. In addition, arterial blood oxygen saturation (SpO2) was monitored with a pulse oximeter (Onyx^®^ Vantage 9590 Finger Pulse Oximeter, Nonin Medical, Inc., Plymouth, MN, USA) and recorded immediately before and immediately after the FT of each leg.

### 2.5. Electromyography (EMG)

Muscle activity during strength and fatigue testing was recorded via a wireless EMG system (Ultium-EMG, Noraxon USA Inc., Scottsdale, AZ, USA). EMG electrodes were placed bilaterally over the rectus femoris, vastus medialis, vastus lateralis, and semitendinosus according to a 3D Muscle Map provided by the EMG software (MR 3.14, myoMUSCLE, Noraxon USA Inc., Scottsdale, AZ, USA) that followed SENIAM guidelines. Site preparation included shaving leg hair and vigorously scrubbing with an alcohol wipe before placing the EMG electrodes (3M Red Dot Monitoring Electrode, Model 2560; 3M Corp., St. Paul, MN, USA; 2 cm between each 1.3 cm effective area). Once the electrodes and wireless transmitters were in place, they were secured with elastic bandages. EMG data were collected at 2000 Hz.

### 2.6. Transcranial Direct Current Stimulation (tDCS)

A tDCS device (Soterix Medical Inc., New York, NY, USA) delivered a small current through two carbon electrodes that were placed inside of two saline-soaked sponges (5 × 7 cm, 35 cm^2^ area; EASYpad, Soterix Medical Inc., New York, NY, USA). The anode was placed over C3 (according to the 10–20 EEG convention), and the cathode was placed over the contralateral supraorbital area. The placement of the anode was selected to unilaterally target the M1 that controls the dominant leg [36,37], and the large size of the electrode also ensured that the leg area of the M1 in the longitudinal fissure (~Cz) was also covered [36]. The sponge electrodes were held in place with an EASYstrap (Soterix Medical Inc., New York, NY, USA) which has ruler-like markings (i.e., cm) that facilitated consistent, individualized electrode placement between sessions. The order of the stimulation (2 mA, 4 mA, or sham) was randomized for sessions 2–4 for each subject. The 2 mA and 4 mA conditions started with a 30 s ramp-up and then remained at the desired intensity for 20 min before a 30 s ramp-down to 0 mA. During the first and last minutes of the sham condition, the intensity was ramped up to 4 mA over 30 s and then immediately ramped down to 0 mA over 30 s. Otherwise, the stimulation intensity remained at 0 mA. The FT on the right leg was performed starting at minute 15 of the 20 min stimulation protocol. Both the right and left FTs were performed during the final 5 min of stimulation [5,6,7,8,38].

To assess the tolerability and blinding of the stimulation, the subjects were asked to report any sensations experienced during the stimulation period and to rate the severity of those sensations on a 10-point Likert scale (1 = “barely perceptible,” 10 = “most I could stand:” [39]). To assess blinding efficacy, the stimulation protocols for each intensity were described to the subjects, and they were asked to guess which intensity they received and to report how confident they were that they guessed correctly on a 10-point Likert scale (1 = “not confident at all,” 10 = “extremely confident” [39]) after each session. The same researcher administered tDCS during each session to all subjects. The subjects and other study personnel were blind to the stimulation order until the last session for a given subject was completed.

### 2.7. Data Analysis

The first two repetitions of the FT were considered adaptations and were removed from all analyses. To examine the effects of tDCS and PPE on leg muscle fatigue, two fatigue indices were calculated for the left and right knee extensors and flexors: a torque-derived fatigue index (FI-T) and a work-derived fatigue index (FI-W). The FI-T was calculated using the peak torque of the relevant repetitions of the FT as follows: ([mean of first five reps—mean of last five reps]/mean of first five reps) * ×100 [6,7,40,41]. A high FI-T indicates that a subject was not able to produce a similar torque during their last five reps compared to their first five reps (i.e., more fatigability). The FI-W was calculated using the total work from the relevant repetitions of the FT as follows: (total work performed in the last half of the FT/total work performed in the first half of the FT) × 100 [6,41]. A low FI-W indicates that a subject was unable to perform a similar amount of work in the second half of the FT compared to the first half (i.e., more fatigability).

The EMG interference signals from each muscle were bandpass filtered (3.5–350 Hz, [5,42]), rectified, smoothed (50 ms root mean square window), and normalized to the highest EMG peak obtained during strength testing. The average muscle activity of the knee extensors (rectus femoris, vastus medialis, vastus lateralis) was calculated to represent the aggregate activity of the knee extensor group. Similar to the FI calculations, the first two repetitions were disregarded as adaptation repetitions, and EMG analyses were performed on the remaining 38 repetitions. To simplify the statistical analysis of the change in EMG activity throughout the FT, the 38 repetitions were grouped into eight windows. The first seven windows contained five sequential, non-overlapping repetitions (window 1 = reps 3–7, window 2 = reps 8–12, etc.), and the last window contained the last three repetitions of the FT. All EMG data were analyzed in the MyoMuscle software (MR3 Version 3, Noraxon USA Inc., Scottsdale, Arizona), and torque/work data were calculated and exported from the HUMAC2015 software (CSMi, Stoughton, MA, USA).

### 2.8. Statistical Analysis

A mixed measures ANOVA with stimulation (sham vs. 2 mA vs. 4 mA) as the within-subject factor and study (the current study (PPE) vs. our previous study (No PPE)) as a between-subject factor was performed on the FI-T and FI-W data. Additionally, to assess potential mechanisms, a repeated measures ANOVA with stimulation (sham vs. 2 mA vs. 4 mA) and time window (1 vs. 2 vs. 3 vs. 4 vs. 5 vs. 6 vs. 7 vs. 8) as within-subject factors was performed on the EMG data in the current (PPE) study. Post hoc comparisons (paired and unpaired *t*-tests) and effect sizes (Cohen’s **d**) were calculated to clarify significant main effects and interactions. Moreover, to examine the effects of PPE on blood oxygen saturation, differences in SpO2 pre- and post-FT were assessed with a paired *t*-test within each stimulation condition. Significance was accepted at *p* < 0.05, and post hoc tests were adjusted with a Bonferroni correction. The normality and sphericity assumptions for the ANOVAs were investigated with the Shapiro–Wilk test and Mauchly’s test of sphericity, respectively. Greenhouse–Geisser corrections were planned and reported when the sphericity assumption was violated. Statistical analyses were performed using GraphPad Prism 9 (GraphPad Software, San Diego, CA, USA).

## 3. Results

All subjects completed the study, and out of the 16 subjects recruited for the current study and 16 subjects from our previous study (No PPE; [6]), none had FI results below the FI bias correction cut-off (FI ≤ 0%; indicating torque production was higher at the end compared to the beginning of the FT). The normality assumption was met for all ANOVAs, but the sphericity assumption for the stimulation factor was not, and the subsequent statistics were Greenhouse–Geisser corrected. Data are reported as mean ± SD in the text, and mean ± SEM in the figures.

**Figure 1:**
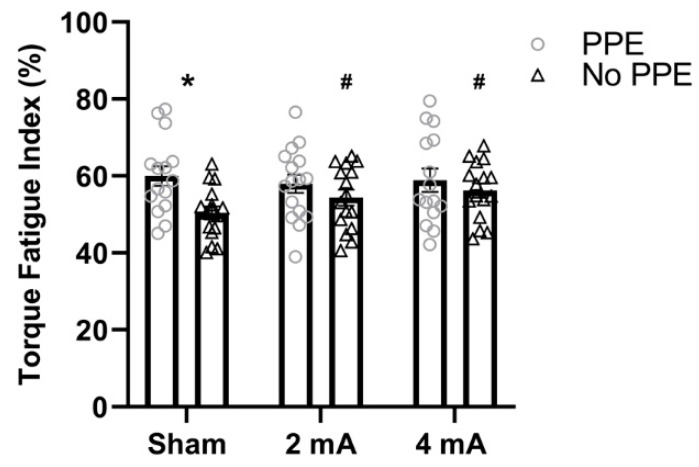
Fatigue index derived from the torque data for the right knee extensors stratified by tDCS condition (sham, 2 mA, and 4 mA) and study (PPE vs. No PPE). * Significantly larger (i.e., increased fatigability) than the No PPE study [6] in the same tDCS condition (sham). ^#^ Significantly larger (i.e., increased fatigability) than sham in the same study (No PPE).

The results of the repeated measures ANOVAs for the torque fatigue index (FI-T) indicate no significant main effect of study for the right knee extensors (F [(1,30) = 3.18, *p* = 0.08, $\eta_{p}^{2}$ = 0.09), right knee flexors (F(1,30) = 1.31, *p* = 0.26, $\eta_{p}^{2}$ = 0.13), left knee extensors (F(1,30) = 2.97, *p* = 0.09, $\eta_{p}^{2}$ = 0.24), and left knee flexors (F(1,30) = 3.69, *p* = 0.06, $\eta_{p}^{2}$ = 0.30). Similarly, there was no significant main effect of stimulation for the right knee extensors (F(1.96,56.81) = 2.67, *p* = 0.08, $\eta_{p}^{2}$ = 0.08), right knee flexors (F(2,60) = 0.32, *p* = 0.73, $\eta_{p}^{2}$ = 0.01), left knee extensors (F(1.67,49.8)= 0.29, *p* = 0.71, $\eta_{p}^{2}$ < 0.01, $\hat{\varepsilon }$ = 0.83), and left knee flexors (F(1.71,51.37) = 1.35, *p* = 0.27, $\eta_{p}^{2}$ = 0.02, $\hat{\varepsilon }$ = 0.86). In addition, the results indicate no significant stimulation × study interactions for the right knee flexors (F(2,60) < 0.00, *p* = 0.99, $\eta_{p}^{2}$ < 0.01), left knee extensors (F(2,60) = 2.17, *p* = 0.12, $\eta_{p}^{2}$ = 0.03), and left knee flexors (F(2,60) = 0.67, *p* = 0.52, $\eta_{p}^{2}$ = 0.01). However, there was a significant stimulation × study interaction for right knee extensors (F(2,58) = 6.65, *p* < 0.01, $\eta_{p}^{2}$ = 0.19). Figure 1 displays the results of the post hoc testing and reveals that, compared to the No PPE study, the subjects in the current PPE study had a significantly higher FI-T (i.e., more fatigability) in the sham condition (PPE: 60 ± 10%, No PPE: 50.4 ± 6.7%; *p* = 0.01, **d** = 1.13), but not in the 2 mA (PPE: 57.9 ± 9.5%, No PPE: 54.4 ± 8.2%; *p* = 0.60, **d** = 0.39) or 4 mA (PPE: 58.9 ± 11.7%, No PPE: 56.4 ± 7.4%; *p* = 0.84, **d** = 0.26) conditions. In the No PPE study (Workman et al. 2020a), both the 2 mA and 4 mA conditions had a significantly higher FI-T compared to sham (*p* = 0.001, **d** = 0.73 and *p* < 0.001, **d** = 1.61, respectively); however, in this PPE study, there were no significant differences in the FI-T in any of the stimulation conditions (sham vs. 2 mA: *p* = 0.67, **d** = 0.21, sham vs. 4 mA: *p* = 0.38, **d** = 0.10, 2 mA vs. 4 mA: *p* = 0.27, **d** = 0.09).

**Figure 2:**
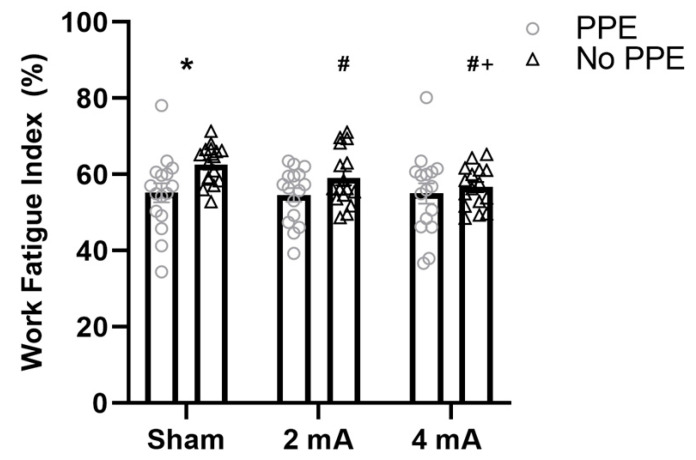
Fatigue index derived from the work data for the right knee extensors stratified by tDCS condition (sham, 2 mA, and 4 mA) and study (PPE vs. No PPE). * Significantly smaller (i.e., increased fatigability) than the No PPE study (Workman et al., 2020 [6]) in the same tDCS condition (sham). ^#^ Significantly smaller (i.e., increased fatigability) than sham in the same study (No PPE). ^+^ Significantly smaller than the 2 mA condition in the same study (No PPE).

The results of the repeated measures ANOVAs for the work fatigue index (FI-W) of the right knee flexors and left knee extensors indicate no significant stimulation × study interactions (F(2,60) = 0.61, *p* = 0.55, $\eta_{p}^{2}$ = 0.01 and F(2,60) = 2.02, *p* = 0.14,$\;\eta_{p}^{2}$ = 0.03, respectively) and no significant main effects of study (F(1,30) = 1.86, *p* = 0.18, $\eta_{p}^{2}$ = 0.22 and F(1,30) = 1.44, *p* = 0.24, $\eta_{p}^{2}$ = 0.20, respectively) or stimulation (F(1.74,52.30) = 0.39, *p* = 0.65, $\eta_{p}^{2}$ = 0.01, $\hat{\varepsilon }$ = 0.87 and F(1.63,48.83) = 1.99, *p* = 0.16, $\eta_{p}^{2}$ = 0.03, $\hat{\varepsilon }$ = 0.81, respectively). However, the results for the left knee flexors indicate a significant main effect of study (F(1,30) = 6.18, *p* = 0.02, $\eta_{p}^{2}$ = 0.48), but no significant main effect of stimulation (F(1.70,51.04) = 0.42, *p* = 0.63, $\eta_{p}^{2}$ = 0.01) or stimulation × study interaction (F(2,60) = 0.13, *p* = 0.88, $\eta_{p}^{2}$ < 0.01). The results of the post hoc test indicate that, collapsed across stimulation conditions, subjects in the PPE study had a significantly lower FI-W compared to the No PPE study (i.e., more fatigability, PPE: 68 ± 0.54, No PPE: 75.4 ± 0.73; *p* < 0.001, **d** = 11.63). In addition, the results of the right knee extensors indicate a significant stimulation × study interaction (F(2,60) = 5.11, *p* = 0.009, $\eta_{p}^{2}$ = 0.08) and main effect of stimulation (F(1.82,54.55) = 6.0, *p* = 0.006, $\eta_{p}^{2}$ = 0.09), but not a main effect of study (F(1,30) = 2.99, *p* = 0.09, $\eta_{p}^{2}$ = 0.39). The post hoc testing for the main effect of stimulation revealed that, collapsed across studies, the FI-W was significantly lower in the 4 mA condition compared to sham (i.e., more fatigability, sham: 58.9 ± 8.6, 4 mA: 55.9 ± 8.4, *p* = 0.001, **d** = 0.36). Figure 2 shows the results of the post hoc testing for the stimulation × study interaction which reveal that, compared to the No PPE study, the subjects in the current PPE study had a significantly lower FI-W (i.e., more fatigability) in the sham condition (PPE: 55.2 ± 10%, No PPE: 62.6 ± 5.1%; *p* = 0.04, **d** = 0.93), but not in the 2 mA (PPE: 54.7 ± 7.3%, No PPE: 59.1 ± 7.4%; *p* = 0.27, **d** = 0.60) or 4 mA (PPE: 55 ± 10.7%, No PPE: 56.7 ± 5.4%; *p* = 0.93, **d** = 0.20) conditions. Moreover, in the No PPE study (Workman et al. 2020), both the 2 mA and 4 mA conditions had a significantly smaller FI-W than sham (*p* = 0.01, **d** = 0.57 and *p* < 0.001, **d** = 1.12, respectively), and 4 mA was significantly smaller than the 2 mA condition (*p* = 0.034, **d** = 0.37). In the current PPE study, however, there were no significant differences in the FI-W between any of the conditions (sham vs. 2 mA: *p* = 0.73, **d** = 0.06, sham vs. 4 mA: *p* = 0.89, **d** = 0.02, 2 mA vs. 4 mA: *p* = 0.81, **d** = 0.04).

**Figure 3:**
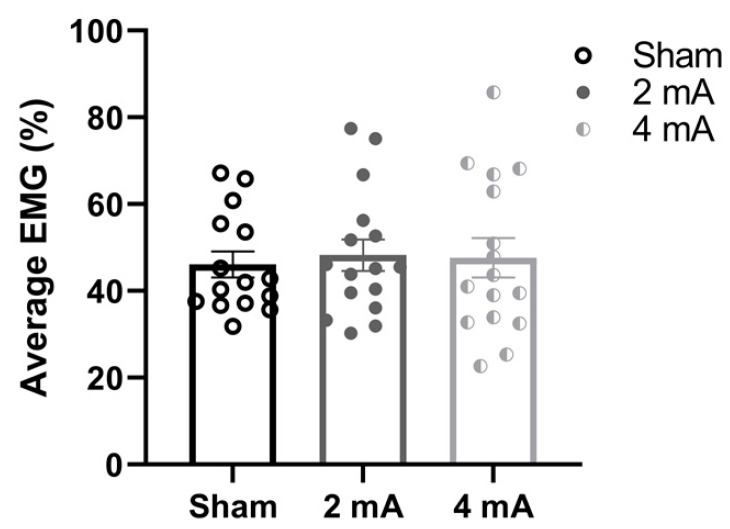
Changes in muscle activity (average EMG%) over the eight time windows of the isokinetic fatigue task, stratified by tDCS condition (sham, 2 mA, 4 mA) in the PPE study.

**Figure 4:**
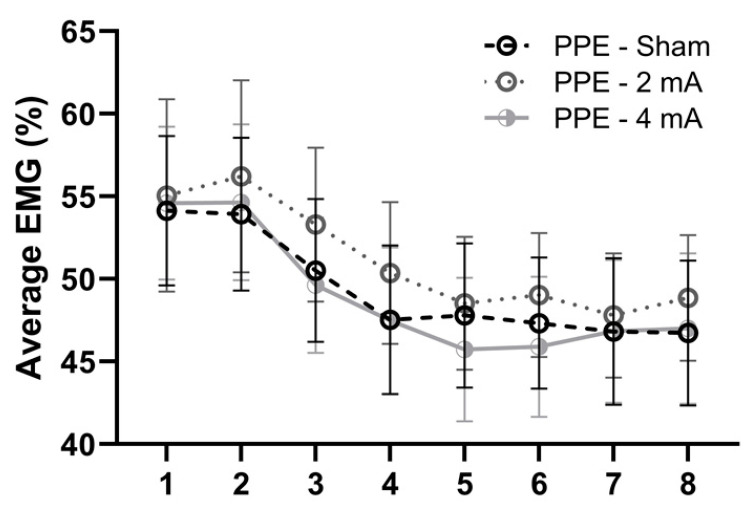
Comparison of the average muscle activity (average EMG %) in the PPE study stratified by tDCS condition (sham, 2 mA, and 4 mA).

Figure 3 shows the change in EMG activity of the right knee extensors, the only muscle group that had a significant interaction (i.e., difference between the studies), over the eight time windows of the FT during each stimulation condition (sham, 2 mA, and 4 mA) in the current PPE study. The repeated measures ANOVA indicated no main effects of window (F(7,120) = 0.69, *p* = 0.68, $\eta_{p}^{2}$ = 0.02), stimulation (F(1.52, 182.2) = 1.76, *p* = 0.18, $\eta_{p}^{2}$ = 0.01, $\hat{\varepsilon }$ = 0.76), or window × stimulation interaction (F(14,240) = 0.08, *p* > 0.99,$\;\eta_{p}^{2}$ < 0.01). Similarly, Figure 4 displays the comparisons of the average EMG activity of the right knee extensors during each tDCS condition in the PPE study. There was no significant effect of stimulation (F(1.87,27.1) = 0.07, *p* = 0.93, $\eta_{p}^{2}$ < 0.00, $\hat{\varepsilon }$ = 0.93).

SpO2 was not significantly different before and after the fatigue test in the sham (pre = 98.73 ± 0.59, post = 98.20 ± 1.15, *p* = 0.06, **d** = 0.58) or 2 mA (pre = 98.88 ± 1.09, post = 98.50 ± 0.82, *p* = 0.08, **d** = 0.39) conditions. However, there was a significant difference in SpO2 before and after the fatigue test in the 4 mA (pre = 98.63 ± 0.81, post = 97.5 ± 1.79, *p* = 0.04, **d** = 0.81) condition.

The most common sensations reported in the three tDCS conditions of the current study were burning (sham: 3.0 ± 1.9; 2 mA: 3.1 ± 1.5; 4 mA: 3.2 ± 1.4), itching (sham: 3.3 ± 0.5; 2 mA: 3.0 ± 1.2; 4 mA: 3.9 ± 1.2), and tingling (sham: 2.3 ± 1.5; 2 mA: 1.8 ± 0.5; 4 mA: 3 ± 1.7) and were all considered mild. For stimulation blinding, 50%, 56.25%, and 43.75% of subjects correctly guessed the sham, 2 mA, and 4 mA conditions, respectively. These tolerability and blinding results are similar to the No PPE study (Workman et al. 2020a) in which burning (sham: 3.3 ± 1.3; 2 mA: 2.3 ± 2.4; 4 mA: 5.2 ± 1.6), itching (sham: 2.3 ± 1.5; 2 mA: 4.3 ± 1.2; 4 mA: 3.6 ± 2.0), and tingling (sham: 1.5 ± 1.0; 2 mA: 2.4 ± 1.1; 4 mA: 3.2 ± 1.6) were the most common sensations experienced, and 68.8%, 62.5%, and 43.8% subjects correctly guessed the stimulation intensity in the sham, 2 mA, and 4 mA conditions, respectively.

## 4. Discussion

To our knowledge, this is the first study that evaluated whether wearing PPE while performing a tDCS-influenced fatigue task affected the main outcomes in healthy young adults. The main finding was that subjects wearing PPE demonstrated significantly increased fatigability (i.e., increased FI-T and decreased FI-W) compared to the No PPE subjects in the sham condition. Moreover, PPE resulted in similar fatigability across all three conditions (sham, 2 mA tDCS, 4 mA tDCS), which contrasts with our previous No PPE study [6] that showed that 2 mA and 4 mA tDCS resulted in greater fatigability compared to sham in a demographically similar population of young, healthy adults. These findings support our hypothesis that wearing PPE would significantly increase the subjects’ fatigue profile. However, given the unique research climate in which this study was conducted (see Methods above), comparing the effects of PPE vs. No PPE in the same subjects (i.e., a within-subject design) was not possible and may decrease the generalizability of the results. Although subjects were carefully matched by sex and age to our previous study (No PPE, [6]) to reduce inter-subject variability inherent to between-subject designs and did not differ in maximal strength performance, these results may be confounded by pre-existing differences between the groups and should be interpreted accordingly.

In our previous study (No PPE, [6]), there was a significant increase in FI-T and decrease in FI-W (i.e., greater fatigability) in the 2 mA and 4 mA conditions compared to sham. However, in the current study, there were no significant differences in FI-T or FI-W in any of the stimulation conditions (sham vs. 2 mA vs. 4 mA). Interestingly, there were only significant differences between the studies in the absence of active tDCS (i.e., sham stimulation). Therefore, these results may indicate that PPE increased fatigue, but these fatiguing effects were not further compounded by tDCS. In other words, a ceiling effect might have already been reached during the sham condition, which then resulted in the null effects of applying 2 mA and 4 mA tDCS and the significant contrast with the No PPE study [6].

It has been proposed that wearing PPE might increase carbon dioxide rebreathing or compromise oxygen consumption, both of which would lead to lower arterial oxygen saturation of hemoglobin [2] and, consequently, increase fatigue. Chandrasekaran et al. (2020) also postulated that face masks might provide resistance to breathing, and evidence from other studies also supports these physiological effects [2]. The blood oxygen saturation (SpO2) results of this study only found pre–post differences in the 4 mA condition. However, these results are not consistent enough to adequately explain the increased fatigability compared to the No PPE study [6]. Thus, our findings agree with the results of Shaw et al. (2020), who also demonstrated a lack of substantial differences between wearing surgical masks, cloth masks, and no face mask conditions on cycle performance (time to exhaustion, peak power), heart rate, blood, and muscle oxygenation [33]. Furthermore, Epstein et al. (2020) measured an increase in end-tidal carbon dioxide at exhaustion while wearing a surgical face mask during a progressive cycle ergometer test [24]. However, this did not affect arterial oxygen saturation, expressed as a percentage of peak power output, during exercise, nor was it detrimental to performance [24]. Thus, these findings support our supposition that none of these potential oxygenation effects of wearing face masks significantly influence performance. However, a limitation of our study was that our pulse oximeter measurement of SpO2 might not be sensitive enough to detect relevant differences. Although a meta-analysis [43] of the accuracy of oxygen saturation measured by pulse oximetry compared to arterial blood samples found a weighted mean correlation coefficient of 0.895, inaccuracies in the equipment of the present study cannot be excluded [44].

EMG activity of the knee extensors was not significantly different between any of the stimulation conditions. Surface EMG has known limitations (e.g., amplitude cancellation, crosstalk, the potential for inconsistent electrode placement between sessions [45,46]) and may not sufficiently indicate changes in the neural drive to the muscles [47]. Thus, EMG may not be adequately sensitive to detect the potentially subtle changes to the central recruitment of spinal motor neurons that ultimately relay the relevant information to the muscles required for task execution. Additionally, the effect of anodal tDCS on torque production may not occur from a postsynaptic effect on cortico-motor projections but could be connected to a presynaptic effect on the motor cortex inter-neuronal network, which would not be detectable with surface EMG. Therefore, other neuromuscular outcomes, such as voluntary activation (VA), potentiated twitch at rest, and motor evoked potentials might provide additional insights beyond EMG [48].

The proposed fatigue model by Rudroff et al., 2016 emphasized the interaction between physiological contributors to fatigue and the environment and task-specific factors [3]. The pandemic has significantly changed the human research environment (environmental dependency; [3]), and these modifications might affect fatigue in humans. Additionally, the self-isolation, lockdowns, and social isolation that have occurred in response to the COVID-19 pandemic may have negative impacts on an individual’s physical and mental capacity [49]. Specifically, anxiety can result in hyperactivation of right and/or hypoactivation of left frontal cortical regions and contribute to negative emotions [50]. Therefore, performing fatiguing experiments under these conditions might alter the results, similar to those presented in this study. Although not directly assessed, it is possible that the sample in the present study might have been affected by the currently challenging, pandemic-altered research environment (i.e., both subjects and researchers wearing PPE). However, additional studies that address these psychological and environmental influences on fatigue are required to verify this speculation.

This study had several limitations. The first was the between-group (PPE study vs. No PPE study) comparison, which might limit the generalizability of the findings. However, the environmental dependency aspect of the Rudroff et al. (2016) fatigue model highlights the importance of the performance environment on fatigue outcomes [3]. Waiting to perform a within-subject comparison post-pandemic (i.e., without the mandated PPE requirements) would not have been representative of this pandemic-specific environment and would have decreased the generalizability of the findings to fatigue studies performed in this same time frame. Additionally, we evaluated a three-layer cloth face mask because this is the standard mask at our institution. Single-layer cloth masks or masks made from other materials which might have yielded different results warrant future investigation. This study evaluated wearing face masks and face shields during a maximal effort isokinetic fatiguing task. Other submaximal tasks that involve longer durations (e.g., ≥ 30 min bouts), such as typical aerobic exercises, may yield different results and should be investigated. In addition, our sample consisted of young, healthy subjects, and the results cannot be extrapolated to other populations. Another limitation of this study is that the blinding protocol may not have been successful, which is common in tDCS research, especially when higher intensities (≥2 mA) are used [51,52,53]. Therefore, more research is needed on ways to improve blinding protocols, such as the use of topical anesthetic creams (e.g., EMLA cream) [54,55] and/or assessing blinding during the stimulation rather than the common end-of-study guess [56]. Lastly, we did not assess environmental changes, psychological stress, or how the participants perceived PPE was affecting their performance (e.g., more difficult breathing), which may have influenced the subjects’ fatigue profiles. Nevertheless, this present study may help provide a reference for future studies aiming to assess the effects of wearing PPE on fatigue in older and in clinical populations. Additionally, including other measures (e.g., voluntary activation, potentiated twitch at rest, and motor evoked potentials) in future investigations might provide other insights into the mechanisms of changes, or lack thereof, from wearing PPE. Moreover, future studies should assess depression and anxiety (e.g., using the Beck Depression and Anxiety Inventories) and evaluate their influence on performance fatigability. Future studies should also consider using continuous-wave near-infrared spectroscopy and/or capillary blood samples to measure blood gasses, pH, electrolytes, and relevant metabolites at baseline and immediately after cessation of a fatiguing task to provide similar and/or additional information to pulse oximetry.

## 5. Conclusions

Fatigability was significantly increased when subjects wore PPE (three-ply face mask and plastic face shield) in the sham condition compared to the No PPE study. PPE, worn by healthy young subjects, yielded similar fatigability across all three tDCS conditions (sham, 2 mA tDCS, 4 mA). This contrasts with our previous pre-pandemic study [6] with no PPE that found increased fatigability from 2 mA and 4 mA tDCS compared to sham. These findings are relevant because PPE use in healthy young adults during tDCS studies might have detrimental effects on performance irrespective of the stimulation intervention. Similar tDCS and other non-invasive brain stimulation studies, in general, especially in clinical populations, are warranted to discover if wearing PPE similarly alters outcomes.

## Figures and Tables

**Table 1:** Subject characteristics. No PPE from Workman et al., 2020 [6]. Data are mean ± SD.

	PPE	No PPE
	(Current Study)	(Workman et al. [6])
N (females)	16 (9)	16 (10)
Age (years)	23.0 ± 2.6	24.5 ± 3.8
Height (cm)	170.2 ± 10.2	170.0 ± 11.7
Weight (kg)	67.3 ± 14.6	71.1 ± 14.4

## Data Availability

Data are available upon request to the corresponding author.

## References

[1] Jefferson T., Del Mar C.B., Dooley L., Ferroni E., Al-Ansary L.A., Bawazeer G.A., van Driel M.L., Nair S., Jones M.A., Thorning S. (2011). Physical interventions to interrupt or reduce the spread of respiratory viruses. Cochrane Database Syst. Rev..

[2] Chandrasekaran B., Fernandes S. (2020). Exercise with facemask; are we handling a devil’s sword? A physiological hypothesis. Med. Hypotheses.

[3] Rudroff T., Kindred J.H., Ketelhut N.B. (2016). Fatigue in multiple sclerosis: Misconceptions and future research directions. Front. Neurol..

[4] Kluger B.M., Krupp L.B., Enoka R.M. (2013). Fatigue and fatigability in neurologic illnesses: Proposal for a unified taxonomy. Neurology.

[5] Workman C.D., Fietsam A.C., Rudroff T. (2020). Different effects of 2 mA and 4 mA transcranial direct current stimulation on muscle activity and torque in a maximal isokinetic fatigue task. Front. Hum. Neurosci..

[6] Workman C.D., Kamholz J., Rudroff T. (2020). Increased leg muscle fatigability during 2 mA and 4 mA transcranial direct current stimulation over the left motor cortex. Exp. Brain Res..

[7] Workman C.D., Kamholz J., Rudroff T. (2019). The tolerability and efficacy of 4 mA transcranial direct current stimulation on leg muscle fatigability. Brain Sci..

[8] Workman C.D., Fietsam A.C., Rudroff T. (2020). Transcranial direct current stimulation at 4 mA induces greater leg muscle fatigability in women compared to men. Brain Sci..

[9] Angius L., Mauger A.R., Hopker J., Pascual-Leone A., Santarnecchi E., Marcora S.M. (2018). Bilateral extracephalic transcranial direct current stimulation improves endurance performance in healthy individuals. Brain Stimul..

[10] Ferrucci R., Vergari M., Cogiamanian F., Bocci T., Ciocca M., Tomasini E., De Riz M., Scarpini E., Priori A. (2014). Transcranial direct current stimulation (tDCS) for fatigue in multiple sclerosis. Neuro Rehabil..

[11] Tecchio F., Cancelli A., Cottone C., Zito G., Pasqualetti P., Ghazaryan A., Rossini P.M., Filippi M.M. (2014). Multiple sclerosis fatigue relief by bilateral somatosensory cortex neuromodulation. J. Neurol..

[12] Lefaucheur J.P., Chalah M.A., Mhalla A., Palm U., Ayache S.S., Mylius V. (2017). The treatment of fatigue by non-invasive brain stimulation. Neurophysiol. Clin..

[13] Fietsam A.C., Workman C.D., Ponto L.L.B., Kamholz J., Rudroff T. (2020). Different effects of transcranial direct current stimulation on leg muscle glucose uptake asymmetry in two women with multiple sclerosis. Brain Sci..

[14] Workman C.D., Fietsam A.C., Rudroff T. (2020). Associations of lower limb joint asymmetry with fatigue and disability in people with multiple sclerosis. Clin. Biomech..

[15] Workman C.D., Kamholz J., Rudroff T. (2020). Transcranial direct current stimulation (tDCS) for the treatment of a multiple sclerosis symptom cluster. Brain Stimul..

[16] Workman C.D., Kamholz J., Rudroff T. (2019). Transcranial direct current stimulation (tDCS) to improve gait in multiple sclerosis: A timing window comparison. Front. Hum. Neurosci..

[17] Cancelli A., Cottone C., Giordani A., Migliore S., Lupoi D., Porcaro C., Mirabella M., Rossini P.M., Filippi M.M., Tecchio F. (2018). Personalized, bilateral whole-body somatosensory cortex stimulation to relieve fatigue in multiple sclerosis. Mult. Scler. J..

[18] Alix-Fages C., Romero-Arenas S., Castro-Alonso M., Colomer-Poveda D., Rio-Rodriguez D., Jerez-Martinez A., Fernandez-Del-Olmo M., Marquez G. (2019). Short-term effects of anodal transcranial direct current stimulation on endurance and maximal force production. A systematic review and meta-analysis. J. Clin. Med..

[19] Angius L., Pageaux B., Hopker J., Marcora S.M., Mauger A.R. (2016). Transcranial direct current stimulation improves isometric time to exhaustion of the knee extensors. Neuroscience.

[20] Radel R., Tempest G., Denis G., Besson P., Zory R. (2017). Extending the limits of force endurance: Stimulation of the motor or the frontal cortex?. Cortex.

[21] Krishnan C., Ranganathan R., Kantak S.S., Dhaher Y.Y., Rymer W.Z. (2014). Anodal transcranial direct current stimulation alters elbow flexor muscle recruitment strategies. Brain Stimul..

[22] Flood A., Waddington G., Keegan R.J., Thompson K.G., Cathcart S. (2017). The effects of elevated pain inhibition on endurance exercise performance. PeerJ.

[23] Muthalib M., Kan B., Nosaka K., Perrey S. (2013). Effects of transcranial direct current stimulation of the motor cortex on prefrontal cortex activation during a neuromuscular fatigue task: An fnirs study. Adv. Exp. Med. Biol..

[24] Epstein D., Korytny A., Isenberg Y., Marcusohn E., Zukermann R., Bishop B., Minha S., Raz A., Miller A. (2021). Return to training in the COVID-19 era: The physiological effects of face masks during exercise. Scand. J. Med. Sci. Sports.

[25] Roberge R.J., Coca A., Williams W.J., Powell J.B., Palmiero A.J. (2010). Physiological impact of the n95 filtering facepiece respirator on healthcare workers. Respir. Care.

[26] Person E., Lemercier C., Royer A., Reychler G. (2018). Effect of a surgical mask on six minute walking distance. Rev. Mal. Respir..

[27] Amann M., Eldridge M.W., Lovering A.T., Stickland M.K., Pegelow D.F., Dempsey J.A. (2006). Arterial oxygenation influences central motor output and exercise performance via effects on peripheral locomotor muscle fatigue in humans. J. Physiol..

[28] Amann M., Romer L.M., Subudhi A.W., Pegelow D.F., Dempsey J.A. (2007). Severity of arterial hypoxaemia affects the relative contributions of peripheral muscle fatigue to exercise performance in healthy humans. J. Physiol..

[29] Baig A.S., Knapp C., Eagan A.E., Radonovich L.J. (2010). Health care workers’ views about respirator use and features that should be included in the next generation of respirators. Am. J. Infect. Control.

[30] Johnson A.T. (2016). Respirator masks protect health but impact performance: A review. J. Biol. Eng..

[31] Perna G., Cuniberti F., Dacco S., Nobile M., Caldirola D. (2020). Impact of respiratory protective devices on respiration: Implications for panic vulnerability during the COVID-19 pandemic. J. Affect. Disord..

[32] Fikenzer S., Uhe T., Lavall D., Rudolph U., Falz R., Busse M., Hepp P., Laufs U. (2020). Effects of surgical and ffp2/n95 face masks on cardiopulmonary exercise capacity. Clin. Res. Cardiol..

[33] Shaw K., Butcher S., Ko J., Zello G.A., Chilibeck P.D. (2020). Wearing of cloth or disposable surgical face masks has no effect on vigorous exercise performance in healthy individuals. Int. J. Environ. Res. Public Health.

[34] Coronavirus in the U.S.: Latest Map and Case Count. https://www.nytimes.com/interactive/2021/us/covid-cases.html.

[35] Jang H., Lee J.Y., Lee K.I., Park K.M. (2017). Are there differences in brain morphology according to handedness?. Brain Behav..

[36] Foerster A.S., Rezaee Z., Paulus W., Nitsche M.A., Dutta A. (2018). Effects of cathode location and the size of anode on anodal transcranial direct current stimulation over the leg motor area in healthy humans. Front. Neurosci..

[37] Ciccone A.B., Deckert J.A., Schlabs C.R., Tilden M.J., Herda T.J., Gallagher P.M., Weir J.P. (2019). Transcranial direct current stimulation of the temporal lobe does not affect high-intensity work capacity. J. Strength Cond. Res..

[38] Martin D.M., Liu R., Alonzo A., Green M., Loo C.K. (2014). Use of transcranial direct current stimulation (tDCS) to enhance cognitive training: Effect of timing of stimulation. Exp. Brain Res..

[39] Workman C.D., Fietsam A.C., Rudroff T. (2020). Tolerability and blinding of transcranial direct current stimulation in people with parkinson’s disease: A critical review. Brain Sci..

[40] Thorstensson A., Karlsson J. (1976). Fatiguability and fibre composition of human skeletal muscle. Acta Physiol. Scand..

[41] Lambert C.P., Archer R.L., Evans W.J. (2001). Muscle strength and fatigue during isokinetic exercise in individuals with multiple sclerosis. Med. Sci. Sports Exerc..

[42] Hameau S., Bensmail D., Roche N., Zory R. (2018). Adaptations of fatigue and fatigability after a short intensive, combined rehabilitation program in patients with multiple sclerosis. J. Rehabil. Med..

[43] Jensen L.A., Onyskiw J.E., Prasad N.G. (1998). Meta-analysis of arterial oxygen saturation monitoring by pulse oximetry in adults. Heart Lung.

[44] Kelly A.M., McAlpine R., Kyle E. (2001). How accurate are pulse oximeters in patients with acute exacerbations of chronic obstructive airways disease?. Respir. Med..

[45] Farina D., Merletti R., Enoka R.M. (2004). The extraction of neural strategies from the surface EMG. J. Appl. Physiol..

[46] Farina D., Merletti R., Enoka R.M. (2014). The extraction of neural strategies from the surface EMG: An update. J. Appl. Physiol..

[47] Del Vecchio A., Negro F., Felici F., Farina D. (2017). Associations between motor unit action potential parameters and surface EMG features. J. Appl. Physiol..

[48] Pageaux B., Angius L., Hopker J.G., Lepers R., Marcora S.M. (2015). Central alterations of neuromuscular function and feedback from group iii-iv muscle afferents following exhaustive high-intensity one-leg dynamic exercise. Am. J. Physiol. Regul. Integr. Comp. Physiol..

[49] Taylor S. (2019). The Psychology of Pandemics: Preparing for the Next Global Outbreak of Infectious Disease.

[50] Stein D.J., Fernandes Medeiros L., Caumo W., Torres I.L. (2020). Transcranial direct current stimulation in patients with anxiety: Current perspectives. Neuropsychiatr. Dis. Treat..

[51] Wallace D., Cooper N.R., Paulmann S., Fitzgerald P.B., Russo R. (2016). Perceived comfort and blinding efficacy in randomised sham-controlled transcranial direct current stimulation (tDCS) trials at 2 mA in young and older healthy adults. PLoS ONE.

[52] O’Connell N.E., Cossar J., Marston L., Wand B.M., Bunce D., Moseley G.L., De Souza L.H. (2012). Rethinking clinical trials of transcranial direct current stimulation: Participant and assessor blinding is inadequate at intensities of 2ma. PLoS ONE.

[53] Fertonani A., Ferrari C., Miniussi C. (2015). What do you feel if i apply transcranial electric stimulation? Safety, sensations and secondary induced effects. Clin. Neurophysiol..

[54] Farnad L., Ghasemian-Shirvan E., Mosayebi-Samani M., Kuo M.F., Nitsche M.A. (2021). Exploring and optimizing the neuroplastic effects of anodal transcranial direct current stimulation over the primary motor cortex of older humans. Brain Stimul..

[55] McFadden J.L., Borckardt J.J., George M.S., Beam W. (2011). Reducing procedural pain and discomfort associated with transcranial direct current stimulation. Brain Stimul..

[56] Turner C., Jackson C., Learmonth G. (2021). Is the “end-of-study guess” a valid measure of sham blinding during transcranial direct current stimulation?. Eur. J. Neurosci..

